# An Improved Kernel Based Extreme Learning Machine for Robot Execution Failures

**DOI:** 10.1155/2014/906546

**Published:** 2014-04-08

**Authors:** Bin Li, Xuewen Rong, Yibin Li

**Affiliations:** ^1^School of Control Science and Engineering, Shandong University, Jinan, Shandong 250061, China; ^2^School of Science, Qilu University of Technology, Jinan, Shandong 250353, China

## Abstract

Robot execution failures prediction (classification) in the robot tasks is a difficult learning problem due to partially corrupted or incomplete measurements of data and unsuitable prediction techniques for this prediction problem with little learning samples. Therefore, how to predict the robot execution failures problem with little (incomplete) or erroneous data deserves more attention in the robot field. For improving the prediction accuracy of robot execution failures, this paper proposes a novel KELM learning algorithm using the particle swarm optimization approach to optimize the parameters of kernel functions of neural networks, which is called the AKELM learning algorithm. The simulation results with the robot execution failures datasets show that, by optimizing the kernel parameters, the proposed algorithm has good generalization performance and outperforms KELM and the other approaches in terms of classification accuracy. Other benchmark problems simulation results also show the efficiency and effectiveness of the proposed algorithm.

## 1. Introduction

The reliability of robot is very important for improving the interactive ability between robot and the changing conditions. In the complex environments in which execution failures can have disastrous consequences for robots and the objects in the surroundings, the prediction ability of robot execution failures is equally important in the robotic field.

However, the prediction of robot execution failures is a difficult learning problem. The first reason is the partially corrupted or incomplete measurements of data. And the second reason is that some prediction techniques are not suitable for predicting the robot execution failures with little samples.

For improving the prediction accuracy of the robot execution failures, in 2009, Twala formulated the robot execution failures problem as a classification task that works with the probabilistic approach-decision tree for handling incomplete data [1]. In 2011, the performance of base-level and meta-level classifiers is compared by Koohi et al. and the Bagged Naïve Bayes is found to perform consistently well across different settings [2]. However, the learning techniques were not incorporated in the aforementioned studies in order to improve the prediction accuracy of robot execution failures. In 2013, Diryag et al. presented a novel method for prediction of robot execution failures based on BP neural networks [3]. The results show that the method can successfully be applied for the robot execution failures with prediction accuracy of 95.4545%. However, it is clear that the learning speed of BP neural networks is generally very slow and may easily converge to local minima. Therefore, some algorithms of machine learning field with better learning performance should be used for the robot execution failures.

The applications of neural networks are very diverse, and, in robotics, many artificial intelligence approaches are applied. Among the approaches of neural networks, extreme learning machine (ELM) proposed by Huang et al. in 2006 has fast learning speed and good generalization performance and has been used in many fields except for the robot execution failures.

The ELM is a learning algorithm for single hidden layer feed-forward neural networks (SLFNs), which determines the initial parameters of input weights and hidden biases randomly with simple kernel function. However, the stability and the generalization performance are influenced by the above learning technique [4]. And some improvements to the ELM learning algorithm have been presented [5].

Among the influence factors of the learning performance of the ELM algorithm, the hidden neurons of the ELM learning algorithm are very important for improving generalization performance and stability of the SLFNs. In [6], we proposed an extreme learning machine with tunable activation function learning algorithm for solving the data dependent on hidden neurons. However, how to choose the suitable combination of activation functions of hidden neurons is still unresolved. In addition, when the feature mapping function of hidden neurons is unknown, kernel function can be used for improving the stability of algorithm [7], which is called the kernel based extreme learning machine (KELM). However, the kernel parameter should be chosen properly for improving the generalization performance of the KELM learning algorithm.

In order to improve the classification accuracy (generalization performance) of robot execution failures, we propose a novel kernel based extreme learning machine in this paper. The kernel parameters of kernel functions of the proposed algorithm are optimized based on the particle swarm optimization approach, which can improve the generalization performance with stable learning process of the proposed algorithm. The simulation results in terms of robot execution failures and some other benchmark problems show the efficiency and effectiveness of the proposed algorithm and are suitable for the robot execution failures problem of little (incomplete) or erroneous data.

The remainder of this paper is organized as follows. The kernel based extreme learning machine (KELM) is introduced in Section 2.  Section 3 describes the particle swarm optimization for KELM learning algorithm. Then, the performance analysis of the proposed algorithm and simulation results of robot execution failures are analyzed in Section 4.  Section 5 gives the performance analysis of the algorithms by two regression and two classification problems. The last section is the conclusions of this paper.

## 2. Kernel Based Extreme Learning Machine

Recently, the ELM learning algorithm with fast learning speed and good generalization performance has been attracting much attention from an increasing number of researchers [4, 7]. In ELM, the initial parameters of hidden layer need not be tuned and almost all nonlinear piecewise continuous functions can be used as the hidden neurons. Therefore, for *N* arbitrary distinct samples {(*x*_*i*_, *t*_*i*_) | *x*_*i*_ ∈ *R* ^*n*^, *t*_*i*_ ∈ *R* ^*m*^, *i* = 1,…, *N*}, the output function in ELM with *L* hidden neurons is

(1) $$\begin{array}{c} f_{L}\left(x\right)=\overset{L}{\underset{i=1}{\sum }}\beta_{i}h_{i}\left(x\right)=h\left(x\right)\beta , \end{array}$$

where *β* = [*β*_1_, *β*_2_,…, *β*_*L*_] is the vector of the output weights between the hidden layer of  *L* neurons and the output neuron and *h*(*x*) = [*h*_1_(*x*), *h*_2_(*x*),…, *h*_*L*_(*x*)] is the output vector of the hidden layer with respect to the input *x*, which maps the data from input space to the ELM feature space [7].

For decreasing the training error and improving the generalization performance of neural networks, the training error and the output weights should be minimized at the same time, that is,

(2) $$\begin{array}{c} \text{Minimize:}\;||H\beta -T||,||\beta ||. \end{array}$$

The least squares solution of (2) based on KKT conditions can be written as

(3) $$\begin{array}{c} \beta =H^{T}{\left(\frac{1}{C}+HH^{T}\right)}^{-1}T, \end{array}$$

where *H* is the hidden layer output matrix, *C* is the regulation coefficient, and *T* is the expected output matrix of samples.

Then, the output function of the ELM learning algorithm is

(4) $$\begin{array}{c} f\left(x\right)=h\left(x\right)H^{T}{\left(\frac{1}{C}+HH^{T}\right)}^{-1}T. \end{array}$$

If the feature mapping *h*(*x*) is unknown and the kernel matrix of ELM based on Mercer's conditions can be defined as follows:

(5) $$\begin{array}{c} M=HH^{T}:m_{ij}=h\left(x_{i}\right)h\left(x_{j}\right)=k\left(x_{i},x_{j}\right), \end{array}$$

thus, the output function *f*(*x*) of the kernel based extreme learning machine (KELM) can be written compactly as

(6) $$\begin{array}{c} f\left(x\right)=\left[k\left(x,x_{1}\right),\ldots ,k\left(x,x_{N}\right)\right]{\left(\frac{1}{C}+M\right)}^{-1}T,\; \end{array}$$

where *M* = *HH* ^*T*^ and *k*(*x*, *y*) is the kernel function of hidden neurons of single hidden layer feed-forward neural networks.

There are many kernel functions satisfying the Mercer condition available from the existing literature, such as linear kernel, polynomial kernel, Gaussian kernel, and exponential kernel. In this paper, we use three typical kernel functions for simulation and performance analysis and the chosen kernel functions are as follows.

(1) Gaussian kernel:

(7) $$\begin{array}{c} k\left(x,y\right)=\exp\left(-a||x-y||\right); \end{array}$$

(2) hyperbolic tangent (sigmoid) kernel:

(8) $$\begin{array}{c} k\left(x,y\right)=\tanh\left(bx^{T}y+c\right); \end{array}$$

(3) wavelet kernel:

(9) $$\begin{array}{c} k\left(x,y\right)=\cos\left(d\frac{||x-y||}{e}\right)\exp\left(-\frac{{||x-y||}^{2}}{f}\right), \end{array}$$

where Gaussian kernel function is a typical local kernel function and tangent kernel function is a typical global nuclear function, respectively [8]. Furthermore, the complex wavelet kernel function is also used for testing the performance of algorithms.

In the above three kernel functions, the adjustable parameters *a*, *b*, *c*, *e*, and *f* play a major role in the performance of neural networks and should be tuned carefully based on the solved problem.

Compared with the ELM learning algorithm, the hidden layer feature mapping need not be known and the number of hidden neurons need not be chosen in the KELM. Moreover, the KELM learning algorithm achieves similar or better generalization performance and is more stable compared to traditional ELM and it is faster than support vector machine (SVM) [7, 9].

## 3. Particle Swarm Optimization for KELM

In KELM learning algorithm, the regulation coefficient *C* and kernel parameters should be chosen appropriately for improving the generalization performance of neural networks. In [7], the parameters are tried in a wide range and are time consuming. And in [10], a hybrid kernel function is proposed for improving the generalization performance of KELM. However, how to choose the optimal value of the parameters of kernel function has not been resolved.

In this paper, an optimization approach is introduced to the KELM for choosing the optimal parameters of kernel function. There are many optimization approaches in machine learning field and, compared with other methods, the particle swarm optimization (PSO) is a biologically inspired computational stochastic optimization technique developed by Eberhart and Kennedy [11]. The PSO approach is becoming popular because of its little memory requiring, simplicity of implementation, and ability to converge to a reasonably optimal solution quickly [12].

Similar to other population based optimization approaches, the PSO algorithm works by initialing the population of individuals randomly in the search space. Each particle of PSO can fly around to find the best solution in the search space; meanwhile, the particles all look at the best solution (best particle) in their path.

Suppose that the dimension of search space of PSO is *D* and the population size is $\hat{N}$. Then, *x*_*i*_^*d*^ and *v*_*i*_^*k*^ are denoted by the current position and the current velocity of *i*th particle at iteration *t*, respectively. Then, the new velocity and position of the particles for the next iteration time are calculated as follows:

(10) vik(t+1)=w·vik(t)+c1·rand()(pik(t)−xik(t)) +c2·rand()(gik(t)−xik(t)),

(11) xik(t+1)=xik(t)+vik(t+1),   1≤i≤N^, 1≤k≤D,

where *p*_*i*_^*k*^ denotes the best position of the *i*th particle during the search process until now, *g*_*i*_^*k*^ represents the global best position, which constitutes the best position found by the entire swarm until now, *w* is the inertia weight, *c*_1_and *c*_2_ are the acceleration constants, and rand() is a random number between 0 and 1.

In PSO algorithm, the inertia weight *w* maintains the expansion ability of exploring new areas in the search space. Therefore, in order to ensure higher exploring ability in the early iteration and fast convergence speed in the last part iteration, the parameter *w* can reduce gradually at the generation increases and is calculated as [13]

(12) $$\begin{array}{c} w\left(t\right)=w_{\max}-\text{iter}\times \frac{\left(w_{\max}-w_{\min}\right)}{\max\;\text{iter}}, \end{array}$$

where *w*_max⁡_ and *w*_min⁡_ are the initial inertial weight and the final inertial weight, respectively. The parameter max⁡  iter is the maximum searching iteration number and iter is the iteration number at the present, respectively.

In addition, in order to enhance the global search in the early part iteration, to encourage the particles to converge to the global optimal solution, and to improve the convergence speed in the final iteration period [12, 14], the acceleration parameters *c*_1_and *c*_2_ are described as

(13) $$\begin{array}{c} c_{1}=\left(c_{1\;\min}-c_{1\;\max}\right)\frac{\text{iter}}{\max\;\text{iter}}+c_{1\;\max}, \end{array}$$

(14) $$\begin{array}{c} c_{2}=\left(c_{2\;\max}-c_{2\;\min}\right)\frac{\text{iter}}{\max\;\text{iter}}+c_{2\;\min}, \end{array}$$

where *c*_1 max⁡_ and *c*_1 min⁡_ are the initial acceleration constant and the final acceleration constant of *c*_1_ and *c*_2 min⁡_ and *c*_2 max⁡_ are the initial acceleration constant and the final acceleration constant of *c*_2_, respectively. Therefore, by changing the acceleration coefficients with time, the cognitive component is reduced and the social component is increased in (10), respectively.

Based on the optimization technology of PSO with self-adaptive parameters *w* and *c*, the parameters of kernel functions of KELM are optimized for improving the generalization performance. Since the number of parameters of kernel functions is different, the dimension of the particle of the proposed algorithm in this paper also changes with the different kernel functions. Therefore, the particle (individual) *θ* of search space in the proposed algorithm can be defined as

(15) $$\begin{array}{c} \theta \in \left[a\right]\;\text{for}\;\text{Gaussian}\;\text{kernel},\\ \theta \in \left[b,c\right]\;\text{for}\;\text{tangent}\;\text{kernel},\\ \theta \in \left[d,e,f\right]\;\text{for}\;\text{wavelet}\;\text{kernel},\;\text{respectively}. \end{array}$$

Thus, the kernel parameter optimization strategy of KELM based on the PSO (which is called the AKELM (adaptive kernel based extreme learning machine) learning algorithm) is summarized as follows.

Given the type of the kernel function, the training set {(*x*_*i*_, *t*_*i*_) | *x*_*i*_ ∈ *R* ^*n*^,  *t*_*i*_ ∈ *R* ^*m*^,  *i* = 1,…, *N*}, and the initial value of regulation coefficient *C*, consider the following steps.

**Step 1**

Initiate the population (particle) based on the kernel function and the velocity and position of each particle.

**Step 2**

Evaluate the fitness function of each particle (the root means standard error for regression problems and the classification accuracy for classification problems).

**Step 3**

According to formulas (10)–(14), the velocity and position of the particle are modified.

**Step 4**

Step 2 and Step 3 are iterated repetitively until the maximal iteration time is satisfied.

**Step 5**

The optimal parameters of kernel function can be determined. Then, based on the optimized parameters, the hidden layer kernel matrix is computed.

**Step 6**

Determine the final output weights *β* in terms of the following equation: *β* = *H* ^*T*^((1/*C*) + *HH* ^*T*^)^−1^ *T*.

## 4. Robot Execution Failures Based on AKELM

In this paper, the AKELM learning algorithm and the KELM algorithm for robot execution failures prediction and the other benchmark problems in machine learning field are conducted in the MATLAB 7.0 with 3.2 GHz CPU and 2G RAM. The number of populations of the PSO for optimizing the kernel parameters is 200 and the maximum iteration number is 100. The initial inertial weights *w*_max⁡_ and *w*_min⁡_ are 0.9 and 0.4, respectively. And the initial acceleration constant values *c*_max⁡_ and *c*_min⁡_ are 2.5 and 0.5, respectively, which means that *c*_1_ changes from 2.5 to 0.5 and *c*_2_ changes from 0.5 to 2.5 over the full range of the search. The regulation coefficient *C* is 100 and the kernel parameters of the KELM learning algorithm are set to 1.

### 4.1. Data Processing

The original robot execution failures data has 90 features, which includes the evolution of forces Fx (15 samples; the following is the same), Fy, and Fz and the evolution of torques Tx, Ty, and Tz measurements on a robot after failure of detection [15].

The robot execution failures problem includes 5 datasets, each of them defining a different learning problem:

- LP1: failures in approach to grasp position,
- LP2: failures in transfer of a part,
- LP3: position of part after a transfer failure,
- LP4: failures in approach to ungrasp position,
- LP5: failures in motion with part.

The feature information and class distribution of the robot execution failures datasets is denoted in Table 1.

As shown from Table 1, the dataset of robot execution failure has small size with 90 features and many classes with 4 for LP1, 5 for LP2, 4 for LP3, 3 for LP4, and 5 for LP5, respectively, which increases the classification difficulty of algorithms.

In [16], a set of five feature transformation strategies was defined for improving the classification accuracy. In the learning of the AKELM and KELM algorithms in neural networks, in order to ensure that different units of data have the same influence on the algorithm, the original data need to be normalized. In this paper, the data is normalized to the interval [−1, +1] and can be described by the following equation:

(16) $$\begin{array}{c} x=2\frac{x-x_{\min}}{x_{\max}-x_{\min}}-1, \end{array}$$

where *x*_max⁡_ and *x*_min⁡_ represent the maximum and minimum values in the original datasets, *x* on the left of the above equation is the original data, and *x* on the right of the above equation is the normalized output data.

For improving the generalization of the robot execution failures data, the positions of samples in each dataset are changed randomly. Then, 90% of samples of the dataset are used for training the neural networks, and the other 10% are testing samples.

### 4.2. Simulation and Performance Analysis

In this study, the performance of the proposed AKELM learning algorithm is compared with the KELM using the robot execution failures data. In the KELM learning algorithm, the learning ability and the generalization performance are influenced mainly by the kernel parameters of different kernel functions. In this paper, the Gaussian kernel function, tangent kernel function, and wavelet kernel function are used to construct different classifier for predicting the robot execution failures.

**Figure 1:**
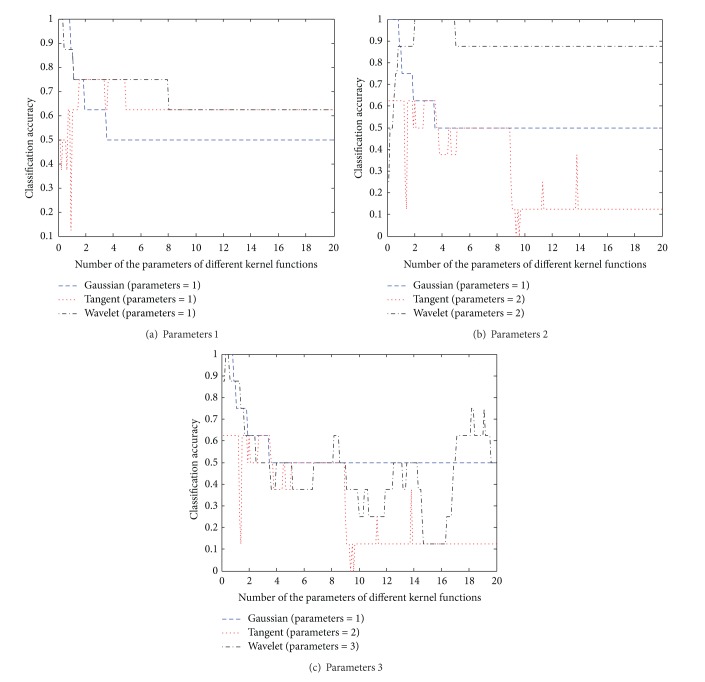
Relationship between the classification accuracy and the number of some parameters of kernel function on LP1 dataset.

Firstly, in order to reduce the search space and accelerate the convergence speed of the PSO algorithm, this paper gives the relationship between the classification accuracy and the number of some parameters of kernel function on robot execution failures using the LP1 dataset. As shown in Figure 1, the classification accuracy in the interval (0,4] has good performance with the difference of the parameters 1 (the values are *a*, *b*, and *d* for Gaussian kernel, tangent kernel, and wavelet kernel, resp.), the parameters 2 (the values are *c* and *e* for tangent kernel and wavelet kernel, resp.), and the parameters 3 (the value is *f* for wavelet kernel). Therefore, the search space of the PSO algorithm is set in the interval between 0 and 4.

Since the simulation results are the same for different running times of the AKELM algorithm and the KELM algorithm, Table 2 shows the comparison of classification results of robot execution failures datasets with three different kernel functions in one running time. As can be seen from the table, the proposed AKELM learning algorithm shows better classification accuracy than the KELM with different kernel functions in most cases and the best classification accuracies are given in boldface. Especially in the LP1 dataset, the proposed algorithm has 100% classification accuracy with Gaussian and wavelet kernel functions, and the generalization performance is better than the best classification approach, Bagged Naïve Bayes in [2], until now to the authors' best knowledge.

## 5. Performance Analysis of AKELM Using Other Benchmark Problems

In this section, the performance of AKELM learning algorithm is compared with the KELM in terms of two regression benchmarks and two classification benchmarks. Specification of the benchmark problems is shown in Table 3. The performance of classification benchmark problems is measured by the classification accuracy and the root mean squares error is used to measure the error of the regression benchmark problems.

Tables 4 and 5 show the performance comparison of AKELM and KELM with Gaussian kernel, tangent kernel, and wavelet kernel neurons; apparently, better test results are given in boldface. The parameters = 1 and parameters = 10 represent the total kernel parameters of different kernel functions set to 1 and 10, respectively.

It can be seen that the proposed AKELM algorithm can always achieve similar or better generalization performance than KELM with different kernel functions and kernel parameters. Moreover, seen from Tables 4 and 5, the KELM learning algorithm with different kernel functions has obviously different generalization performance. However, the proposed AKELM learning algorithm has similar generalization performance to different kernel functions, which means that the proposed algorithm has stable performance with kernel parameters optimized by means of the PSO algorithm, although searching the optimal parameters needs some time as the training time shown in Tables 4 and 5.

## 6. Conclusions

In this study, a novel learning algorithm AKELM has been developed based on the KELM learning algorithm and the PSO approach with self-adaptive parameters. In the proposed AKELM learning algorithm, the parameters of kernel functions of neural networks are adjusted for searching the optimal values by the PSO algorithm.

As shown from the simulation results, the generalization performance of the proposed algorithm in terms of the robot execution failures datasets was found to be significantly improved compared to the KELM learning algorithm. And the other benchmark of regression and classification problems also shows that the proposed algorithm can achieve better generalization performance and has more stable ability than KELM algorithm.

## Figures and Tables

**Table 1:** Feature information and class distribution of the robot execution failures.

Datasets	Instances	Classes
LP1	88	4 (1 = 24%; 2 = 19%; 3 = 18%; 4 = 39%)
LP2	47	5 (1 = 43%; 2 = 13%; 3 = 15%; 4 = 11%; 5 = 19%)
LP3	47	4 (1 = 43%; 2 = 19%; 3 = 32%; 4 = 6%)
LP4	117	3 (1 = 21%; 2 = 62%; 3 = 18%)
LP5	164	5 (1 = 27%; 2 = 16%; 3 = 13%; 4 = 29%; 5 = 16%)

**Table 2:** Classification accuracy of robot execution failures based on KELM and AKELM algorithms.

Kernel data	Gaussian		Tangent		Wavelet	
	KELM	AKELM	KELM	AKELM	KELM	AKELM
LP1	**100%**	**100%**	62.50%	**87.50%**	**100%**	**100%**
LP2	57.14%	57.14%	57.14%	**85.71%**	57.14%	**85.71%**
LP3	57.14%	**71.43%**	57.14%	**85.71%**	57.14%	**100%**
LP4	75%	**100%**	75%	**83.33%**	83.33%	**100%**
LP5	57.14%	**64.29%**	0%	**78.57%**	50%	**71.43%**

**Table 3:** Specification of benchmarks of regression and classification problems.

Datasets	Names	Attributes	Classes	Training data	Testing data
Regression	Box and Jenkins gas furnace data	10	1	200	90
	Auto-Mpg	7	1	320	78

Classification	Wine	13	3	150	28
	Diabetes	8	2	576	192

**Table 4:** Comparison of performance by AKELM and KELM learning algorithms for the regression problems.

Algorithms with different kernel functions	Box and Jenkins gas furnace data			Auto-Mpg		
	Training error	Testing error	Training time (seconds)	Training error	Testing error	Training time (seconds)
KELM (parameters = 1, Gaussian)	0.0120	0.0188	0.0394	0.0529	0.0599	0.1213
KELM (parameters = 1, tangent)	0.0627	0.0655	0.0116	0.6680	0.7756	0.0346
KELM (parameters = 1, wavelet)	0.0121	0.0206	0.0177	0.0509	**0.0597**	0.0415
KELM (parameters = 10, Gaussian)	0.0183	0.0213	0.0149	0.0685	0.0732	0.0286
KELM (parameters = 10, tangent)	0.2245	0.1986	0.0044	0.2071	0.2085	0.0261
KELM (parameters = 10, wavelet)	0.0306	0.0382	0.0101	0.0662	0.0712	0.0360
AKELM (Gaussian)	0.0133	**0.0183**	26.1250	0.0503	**0.0597**	74.7656
AKELM (tangent)	0.0223	**0.0242**	25.2500	0.0735	**0.0735**	73.8906
AKELM (wavelet)	0.0133	**0.0183**	28.3906	0.0502	**0.0597**	84.9688

**Table 5:** Comparison of performance by AKELM and KELM learning algorithms for the classification problems.

Algorithms with different kernel functions	Wine			Diabetes		
	Training accuracy	Testing accuracy	Training time (seconds)	Training accuracy	Testing accuracy	Training time (seconds)
KELM (parameters = 1, Gaussian)	100%	**100%**	0.0277	84.38%	77.08%	0.1394
KELM (parameters = 1, tangent)	51.33%	50%	0.0067	73.78%	73.44%	0.1326
KELM (parameters = 1, wavelet)	100%	**100%**	0.0070	86.81%	76.56%	0.1347
KELM (parameters = 10, Gaussian)	100%	**100%**	0.0083	78.99%	79.17%	0.0919
KELM (parameters = 10, tangent)	39.33%	42.86%	0.0023	65.80%	65.63%	0.0904
KELM (parameters = 10, wavelet)	100%	96.43%	0.0061	80.03%	77.08%	0.1361
AKELM (Gaussian)	100%	**100%**	17.8594	90.45%	**80.21%**	260.7031
AKELM (tangent)	97.33%	**100%**	13.9375	73.26%	**79.17%**	313.8750
AKELM (wavelet)	100%	**100%**	16	89.06%	**79.69%**	335.5469
